# Supplementary material for: Causal Relationship between Aging and Anorexia Nervosa: A White-Matter-Microstructure-Mediated Mendelian Randomization Analysis
Source: Biomedicines. 2024 Aug 16;12(8):1874. doi: 10.3390/biomedicines12081874 (PMC11351342; doi:10.3390/biomedicines12081874)
Supplement: Supplementary file 1 [file biomedicines-12-01874-s001.zip › Supplementary Figure/Figure S4/Singe SNP plot of MR analysis for TL to ubm-b-1702.pdf]

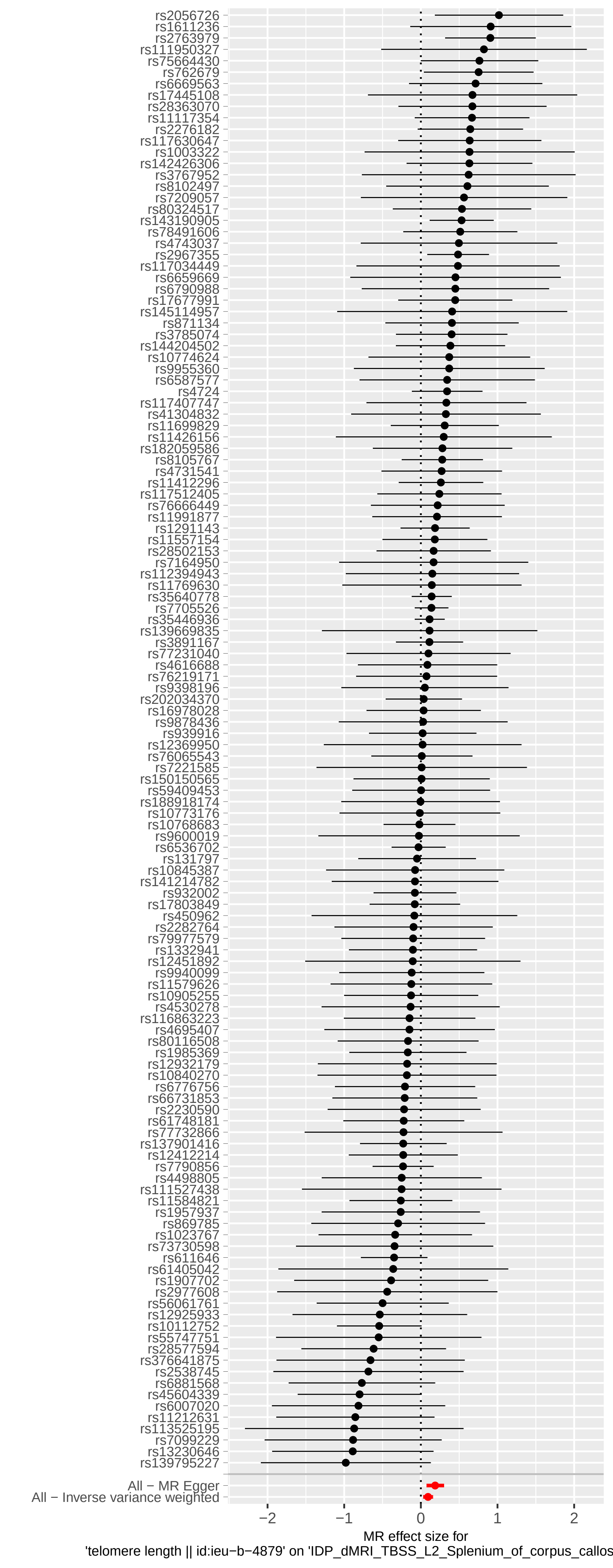
rs2056726
rs1611236
rs2763979
rs111950327
rs75664430
rs762679
rs6669563
rs17445108
rs28363070
rs11117354
rs2276182
rs117630647
rs1003322
rs142426306
rs3767952
rs8102497
rs7209057
rs80324517
rs143190905
rs78491606
rs4743037
rs2967355
rs117034449
rs6659669
rs6790988
rs17677991
rs145114957
rs871134
rs3785074
rs144204502
rs10774624
rs9955360
rs6587577
rs4724
rs117407747
rs41304832
rs11699829
rs11426156
rs182059586
rs8105767
rs4731541
rs11412296
rs117512405
rs76666449
rs11991877
rs1291143
rs11557154
rs28502153
rs7164950
rs112394943
rs11769630
rs35640778
rs7705526
rs35446936
rs139669835
rs3891167
rs77231040
rs4616688
rs76219171
rs9398196
rs202034370
rs16978028
rs9878436
rs939916
rs12369950
rs76065543
rs7221585
rs150150565
rs59409453
rs188918174
rs10773176
rs10768683
rs9600019
rs6536702
rs131797
rs10845387
rs141214782
rs932002
rs17803849
rs450962
rs2282764
rs79977579
rs1332941
rs12451892
rs9940099
rs11579626
rs10905255
rs4530278
rs116863223
rs4695407
rs80116508
rs1985369
rs12932179
rs10840270
rs6776756
rs66731853
rs2230590
rs61748181
rs77732866
rs137901416
rs12412214
rs7790856
rs4498805
rs111527438
rs11584821
rs1957937
rs869785
rs1023767
rs73730598
rs611646
rs61405042
rs1907702
rs2977608
rs56061761
rs12925933
rs10112752
rs55747751
rs28577594
rs376641875
rs2538745
rs6881568
rs45604339
rs6007020
rs11212631
rs113525195
rs7099229
rs13230646
rs139795227
All − MR Egger
All − Inverse variance weighted
−2
−1
0
1
2
MR effect size for
'telomere length || id:ieu−b−4879' on 'IDP_dMRI_TBSS_L2_Splenium_of_corpus_callos
